# Resin defense in *Pinus–Fusarium circinatum* interactions: an evolutionary paradox

**DOI:** 10.1093/jxb/erag095

**Published:** 2026-04-15

**Authors:** Tshepo J Mmushi, Francinah M Ratsoma, Thabiso E Motaung

**Affiliations:** Division of Microbiology, Department of Biochemistry, Genetics, and Microbiology, Forestry and Agricultural Biotechnology Institute, University of Pretoria, Private Bag X20, Hatfield, 0028, Pretoria, South Africa; Division of Microbiology, Department of Biochemistry, Genetics, and Microbiology, Forestry and Agricultural Biotechnology Institute, University of Pretoria, Private Bag X20, Hatfield, 0028, Pretoria, South Africa; Division of Microbiology, Department of Biochemistry, Genetics, and Microbiology, Forestry and Agricultural Biotechnology Institute, University of Pretoria, Private Bag X20, Hatfield, 0028, Pretoria, South Africa

**Keywords:** Chemical defense response, conifers, *Fusarium circinatum*, *Pinus* spp, resin ducts, terpenes

## Abstract

This article comments on:

**Fariña-Flores D, Fernández de Simón B, Hernández-Escribano L, Robertson L, Morales Clemente MT, Conde M, Iturritxa E, Raposo R.** 2026. Resin-based response of *Pinus pinaster* and *P. radiata* during infection by *Fusarium circinatum*. Journal of Experimental Botany **77**, 2490–2505. https://doi.org/10.1093/jxb/erag023

This article comments on:

**Fariña-Flores D, Fernández de Simón B, Hernández-Escribano L, Robertson L, Morales Clemente MT, Conde M, Iturritxa E, Raposo R.** 2026. Resin-based response of *Pinus pinaster* and *P. radiata* during infection by *Fusarium circinatum*. Journal of Experimental Botany **77**, 2490–2505. https://doi.org/10.1093/jxb/erag023

**Resin has long been a vital part of conifer defense, providing both physical and chemical protection against pests and pathogens. This assumption gives rise to an evolutionary paradox: why do some pine species that produce more resin and larger resin ducts remain vulnerable to disease? Fariña-Flores *et al.* (2026) examined this question by comparing the response of resistant *Pinus pinaster* and susceptible *P. radiata* following infection with *Fusarium circinatum.* Resin-based defences are generally considered protective; however, their results suggest that under certain conditions they may be associated with increased vulnerability rather than consistent effective resistance.**

## Insights into resin-based defense

In response to biotic (e.g. fungal inoculations and insects) and abiotic stress (e.g. mechanical wounding and temperature), *Pinus* species synthesize copious amounts of terpene-rich resins (da Silva Rodrigues-Honda *et al.*, 2025). In this issue of the *Journal of Experimental Botany* (Fariña-Flores *et al.*, 2026) demonstrate that resin production in conifers depends more on the timing and mode of mobilization than on the total amount of resin produced in response to fungal infection or wounding. *Pinus* species contain a complex chemical defense network of ∼174 terpenoids, which predominantly include monoterpenes, sesquiterpenes, and diterpene resin acids. Furthermore, *Pinus* terpene biosynthesis is associated with vascular tissues through the classical mevalonic acid pathway (MVA) in the cytosol or the 2-C-methyl-D-erythritol 4-phosphate pathway (MEP) through the plastids (Bohlmann and Keeling, 2008).

Resin ducts are long tubes found between cells that are covered with epithelial cells that produce oleoresin, which is a complex mixture of monoterpenes, sesquiterpenes, and diterpene acids (Phillips and Croteau, 1999). These resins seal wounds, deter insects, and inhibit fungal growth through both physical blockage and chemical toxicity. There are two types of ducts, namely constitutive ducts, formed during normal development, and traumatic ducts, induced by wounding or infection (Celedon and Bohlmann, 2019). Terpenoids within resin act as bioactive defense compounds, with their spectrum shaped by species genetics and environmental factors. Recent studies suggest that resin composition and duct density respond to hormonal signals involving jasmonate and ethylene (Hernandez-Escribano *et al.*, 2020; López-Goldar *et al.*, 2020). When pathogens such as *Fusarium circinatum* develop tolerance to resin compounds or exploit duct structures, the defense system can become a pathway for infection (Fariña-Flores *et al.*, 2026).

For instance, *P. pinaster* shows a concerted stimulation of the MVA pathway and an early accumulation of antimicrobial sesquiterpenes and diterpenes (Hernandez-Escribano *et al.*, 2020; López-Goldar *et al.*, 2020), consistent with its recognized resistance to *F. circinatum* (Hodge and Dvorak, 2000; Elvira-Recuenco *et al.,* 2014). In contrast, the more susceptible *P. radiata* (Hodge and Dvorak, 2000; Iturritxa *et al.*, 2012) displays a delayed induction of terpene biosynthesis, with monoterpenes accumulating at later infection stages (Visser *et al.*, 2018). These responses suggest that the timing and composition of resin deployment, rather than the total resin content, are closely associated with reduced lesion development and pathogen progression (Fariña-Flores *et al.*, 2026).

Anatomical analyses further revealed that *P. radiata* produced larger and more resin ducts than *P. pinaster,* yet sustained greater lesion expansion and hyphal colonization (Fariña-Flores *et al.*, 2026). Microscopic observations showed the presence of *F. circinatum* hyphae within resin canals, supporting the interpretation that these structures can, under certain conditions, be involved in host colonization rather than functioning solely as defensive barriers (Martín-Rodrigues *et al.*, 2013). The findings suggest that resin ducts, although evolved as defense structures, may also act as a channel for infection, an evolutionary inversion reported in other specialized host–pathogen systems (Celedon and Bohlmann, 2019).

The multi-layered approach by Fariña-Flores *et al.* (2026) shows that the timing of defense activation affects the response and that pathogens can manipulate host signals to their benefit. These findings align with previous research indicating that early hormonal signaling, especially involving jasmonate and ethylene, is vital for resistance to necrotrophic pathogens (Visser *et al.*, 2018; Hernandez-Escribano *et al.*, 2020). Fungal interference with jasmonate and ethylene may cause excessive resin duct formation and divert carbon towards non-protective resin (Wasternack and Strnad, 2019). The study also supports reports that certain *Fusarium* species can tolerate or metabolize host terpenes (Blomquist *et al.*, 2010; Slinski *et al.*, 2015). Overall, this is a demonstration that co-evolved pathogens can undermine plant defenses through both metabolic and anatomical strategies. By combining metabolomic and gene expression data, Fariña-Flores *et al*. (2026) show that chemical defense is dynamically controlled through transcriptional and anatomical feedback mechanisms.

In addition to the quantity, the composition of resin greatly influences defensive effectiveness. *Pinus pinaster* has elevated baseline concentrations of sesquiterpenes and diterpenes, while *P. radiata* has generated more volatile monoterpenes such as citronellal, which could be ineffective antifungal agents (Fariña-Flores *et al.*, 2026). These phenotypical profiles were consistent with those observed in the resistant *P. tecunumanii* pine (Visser *et al.*, 2018) and in Austrian pine systems in which terpenoids mediate systemic induced resistance (Ghosh *et al.*, 2024). After infection, *P. pinaster* up-regulated sesquiterpenes and diterpenes early on, whereas *P. radiata* responded later through monoterpene accumulation.

**Fig. 1.**
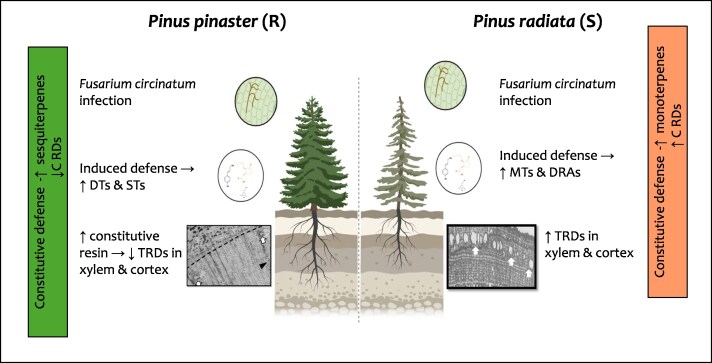
Schematic representation of resin biosynthesis of resistant and susceptible *Pinus* seedlings in response to *Fusarium circinatum*. Resistant (*Pinus pinaster*) and susceptible (*Pinus radiata*) hosts differ in defense timing, terpene composition, and resin duct responses. *P. pinaster* has a higher constitutive resin content enriched in STs and a smaller resin duct system. In contrast, *P. radiata* is enriched in MTs and has larger resin ducts. Upon infection with *F. circinatum,* resistant seedlings show early induced accumulation of STs and DTs, with limited induction of TRDs, whereas susceptible seedlings show delayed defense responses, increased MTs and DRAs, and increased TRD formation in both xylem and cortex (Fariña-Flores *et al.*, 2026). Constitutive resin profiles represent baseline differences between species, whereas changes in terpene composition and TRD formation occur only following pathogen infection. Arrows (→) indicate causal relationships; ↑, increase; ↓, decrease. R, disease resistant; S, disease susceptible; MTs, monoterpenes; DTs, neutral diterpenes; STs, sesquiterpenes; DRAs, diterpene resin acids; CRDs, constitutive resin ducts; TRDs, traumatic resin ducts.

Transcriptomic data also revealed enhanced activation of the MVA pathway in *P. pinaster*, as expected for early signaling in resistant genotypes (Visser *et al.*, 2018; Hernandez-Escribano *et al.*, 2020). Fariña-Flores *et al.* (2026) thus expand our understanding of how chemical diversity and timing jointly determine outcomes in resin-based defense (Fig. 1).

Resin duct formation and terpene biosynthesis demand significant metabolic costs by conifers (Phillips and Croteau, 1999). Fariña-Flores *et al.* (2026) show that this investment does not necessarily translate into increased resistance when pathogens are able to tolerate resin chemistry or utilize resin ducts during colonization. These findings highlight an evolutionary paradox and challenge the common assumption that high resin yield or larger duct systems are reliable indicators of disease resistance. Instead, defense effectiveness appears to depend on regulatory efficiency, including timing and coordination of chemical and anatomical responses. From a forestry and breeding perspective, resin quantity or duct size alone should not be considered as an exclusive selection criterion. Although high resin productivity may help defend against insects, it may also increase susceptibility to pathogens that can detoxify terpenoids or exploit ducts as invasion routes. Future breeding efforts should thus focus on regulatory efficiency, particularly genes involved in early hormonal signaling and balanced duct induction (Hernandez-Escribano *et al*., 2020; Zamora-Ballesteros *et al*., 2021).

The study further emphasizes the importance of defense plasticity in the context of climate change. Increases in temperature and drought conditions alter terpene volatility and resin flow (Celedon and Bohlmann, 2019), potentially shifting host–pathogen dynamics. Integrating molecular data with remote sensing and forest surveillance may facilitate the prediction of resin–pathogen interactions at the landscape scale.

## Future directions in conifer defense biology

The study by Fariña-Flores *et al*. (2026) has set a benchmark for integrative defense research. Building on this foundation, future studies should aim to identify fungal enzymes responsible for terpene detoxification and clarify how pathogens manipulate host signaling. Moreover, combining transcriptomic and metabolomic approaches may help improve forest biocontrol strategies while also identifying early biomarkers for resistance screening. In addition, hormonal crosstalk remains a promising area for future research. Specifically, both resin duct formation and terpene biosynthesis are regulated by interactions between the jasmonate, salicylate, and ethylene pathways (Hernandez-Escribano *et al*., 2020), highlighting intricate regulatory mechanisms worth exploring further. Recognizing the fine-tuning of these networks in resistant trees will show why some species develop rapid, controlled defense, while other species overinvest and become vulnerable. Incorporating climate-related variables into these frameworks will enhance predictions of defense stability under environmental stress. Ultimately, this research redefines resin as a dynamic interface between ecology and biochemistry. The efficiency and timing of resin production are more critical for effective defense than the volume produced. Understanding the relationship between resin quantity and quality helps us better understand tree immunity and underscores the importance of integrated approaches in forest health research (Fariña-Flores *et al.*, 2026).
